# Going beyond Mindfulness: How Concentration and Tranquility Commonly Co-Arising with Mindfulness Account for Mental Health

**DOI:** 10.3390/ijerph20085470

**Published:** 2023-04-11

**Authors:** Ryan M. K. Chan, Winnie W. S. Mak, Ben C. L. Yu

**Affiliations:** Department of Psychology, The Chinese University of Hong Kong, Hong Kong SAR, China; chanryanmk@gmail.com (R.M.K.C.); clyuben@gmail.com (B.C.L.Y.)

**Keywords:** concentration, tranquility, mindfulness, nonattachment, mental health, samadhi, calmness, stillness, stress, distress

## Abstract

Concentration and tranquility usually co-arise with mindfulness during mindfulness practice and in daily life and may potentially contribute to mental health; however, they have rarely been studied in empirical research. The present study aimed to examine the relationship of concentration and tranquility with mindfulness and indicators of mental health. With no existing self-report measure assessing concentration and tranquility, the Concentration Scale and Tranquility Scale were first developed and validated. Items were developed based on the extant literature, rated by a group of experts, and selected according to their ratings. Exploratory factor analyses (EFA; *n* = 384) and confirmatory factor analyses (CFA; *n* = 384) were employed in separate samples of university students and community adults to establish the factor structure of both scales. Their construct validity was established in another similar sample (*n* = 333) by examining their correlations with variables including (a) concentration-related concepts, (b) tranquility-related concepts, (c) mindfulness-related concepts, and (d) perceived stress and psychological distress. The relationships between concentration, tranquility, mindfulness, perceived stress and psychological distress were then examined by hierarchical multiple regressions, both cross-sectionally and longitudinally. A single-factor structure was found by the EFA and confirmed by the CFA for both scales. Concentration and tranquility were significantly and positively associated with (a) attentional control and (b) mindfulness and nonattachment; and negatively associated with (c) irritability and (d) perceived stress and psychological distress. Concentration and tranquility were found to have a significant incremental value over the effect of mindfulness on indicators of mental health. Concentration and tranquility can incrementally explain mental health above and beyond the effect of mindfulness.

## 1. Introduction

Research has found that mindfulness-based programs improve physical and mental health [1], and such improvements have been observed for a range of psychological and medical conditions [2]. Studies have also been conducted to investigate the underlying mechanisms of mindfulness-based programs and mediators leading to mental health in mindfulness-based programs. The findings of these have shown that in addition to mindfulness [3], nonattachment [4], self-compassion [5], and cognitive and emotional reactivity [3] can mediate and explain the effects on mental health outcomes above and beyond mindfulness. However, other psychological qualities that commonly arise in mindfulness-based programs, namely concentration and tranquility, might not have been fully considered in these empirical investigations.

During mindfulness-based programs, participants may experience concentration of the mind and a sense of tranquility simultaneously [6,7]. However, concentration and tranquility are rarely studied empirically and have not been explicitly identified in the curricula of mindfulness-based programs. Without proper understanding of these qualities, participants of mindfulness-based programs may mix them up with mindfulness. For example, some may mistakenly believe that if they are not concentrated and their mind frequently wanders off from the target objects, they are not mindful; others may misunderstand that if they do not become calm, they must have failed the mindfulness practice. In fact, concentration and tranquility are psychological constructs that are distinct from mindfulness.

Concentration refers to the one-pointedness [8,9] and unification of the mind [10,11], which has the characteristic of non-wandering or non-distraction [11,12], with one’s attention being unbroken, sustained, focused, and fixed on a single object [9,10]. Examples of common objects include the breath, a visualization, the mind, the present moment [13], or other objects in daily life, such as music [14]. In Western psychology, concentration is generally seen as one aspect of attention, sometimes discussed in terms of focused attention, controlled attention, sustained attention, or vigilance, though these terms usually refer more to one’s readiness and/or ability to detect a critical signal rather than one’s skill in maintaining the desired focus of attention [14].

Concentration is also akin to selective attention [15], which is defined as the ability to focus on one object and ignore all others [16]. An unconcentrated mind wanders to other things [17], moves in a scattered manner, and cannot stay focused but rushes from idea to idea and from thought to thought, without inner control [9]. Such a distracted mind can be overwhelmed by worries and concerns, and it may see things only in fragments and be distorted by ripples of random thoughts [9]. Based on the above descriptions and conceptualizations, concentration is operationally defined in the present study as “one-pointedness of the mind enabling one to have unbroken and sustained attention focusing on a single object”.

Concentration is considered to exist naturally in the mind [18], be present in all types of consciousness [12], and be possessed by everyone [8]. It arises not only during meditation but also in daily life. It can be developed in stages with different levels of intensity, ranging from the ordinary level of concentration to the deepest level of absorption [11]. The ordinary level of concentration is the level of concentration that occurs in daily life and varies from one person to another, since each person has different degrees of natural concentration ability [11].

Concentration can be cultivated through concentrative practice/meditation (also named samatha or focused-attention practice/meditation) [15,19]. It commonly arises in concentrative meditation in which sustained attention is developed by attending to just one selected object with the total exclusion of all other objects and experiences [15,20]. Consistent with historical accounts of meditation training that usually starts with concentrative meditation [21,22], most mindfulness-based programs begin with concentrative meditation. For example, in the early stages of meditation practice in the Mindfulness-based Stress Reduction (MBSR) program and the Mindfulness-based Cognitive Therapy (MBCT) program, participants are invited to practice breath meditation by focusing their attention only on the flow of their breath and repeatedly bringing their attention back to their breath each time the mind wanders off in order to cultivate concentration [6,7].

Research on concentrative practice has shown that concentration improves both physical and mental health. It increases the activity of the parasympathetic system to reduce stress responses [23], improves the efficiency of gas exchange in the lungs [24], increases cognitive flexibility [25], and alleviates anxiety and stress [26]. Since concentrative practice is often embedded in mindfulness-based programs without being explicitly identified, concentration and mindfulness might be misunderstood by lay practitioners of mindfulness-based programs to be the same. As noted by Mikulas [14], Western psychologists “know surprisingly little about the development of concentration”, and concentration is “very often confused and confounded” with mindfulness [14]. Many research studies have reported how mindfulness helps people to quiet their minds and relax into the here and now which may be primarily due to the development of concentration, often unintentionally [14]. Thus, the reported effects of mindfulness-based programs may possibly be due to concentration instead of or in addition to mindfulness.

Mindfulness is in fact different from concentration. A widely-adopted operational definition of mindfulness in contemporary psychology is that of Kabat-Zinn [27], who defined it as “the awareness that emerges through paying attention on purpose, in the present moment, and nonjudgmentally to the unfolding of experience moment by moment” [27]. Another commonly accepted definition describes mindfulness as “a kind of nonelaborative, non-judgmental, present-centered awareness in which each thought, feeling, or sensation that arises in the attentional field is acknowledged and accepted as it is” [28]. In Buddhism’s canonical sources, mindfulness is described as “a quality of lucid awareness that allows the object to stand forth with a vivid and distinct presence” [29]. What is consistent among these definitions is that mindfulness is the awareness of target object(s). The target object(s) can be “the unfolding of experience” [27], a “single object or a continuum of interrelated events” [19], a “thought, feeling, or sensation that arises in the attentional field” [28], or an “action” [30]. In Western psychology, awareness is commonly conflated with attention even though they involve distinct brain processes [31]. Similarly, the nature of mindfulness as “awareness” is often confused with the characteristic of concentration as “sustained attention”.

Although mindfulness and concentration are two distinct concepts, they have been proposed to work closely, with distinct but complementary roles in practice [18], leading to mental well-being [29,32]. Scholars have suggested that concentration unifies and harmonizes the energies of the mind [33], enhances one’s selective ability for pre-attentive orienting, and creates a calm and focused state to stabilize the mind [18], thereby providing an optimal state for mindfulness to perform its functions effectively. On the other hand, mindfulness is proposed to regulate the breadth and intensity of one’s concentration [34], expand one’s scope of attention, and bring experiential objects into the focus of one’s field of awareness [18,29]. It further retains information and integrates various aspects of the perceptual process so that one becomes aware of the characteristics of moment-to-moment experienced phenomena, supporting the direct realization and understanding of the arising and passing of phenomena [18,29]. Concentration and mindfulness, when cultivated together, have been suggested to improve mental health [30] by increasing adaptive and decreasing maladaptive thoughts, feelings, and behaviors [20].

Tranquility is also closely related to and commonly co-arises with concentration and mindfulness in mindfulness practice and in daily life. It has been proposed that when concentration arises in the mind, the one-pointedness of the mind subdues the wavering of the mind [12], and the mind becomes more and more tranquil and calm [9,35]. Tranquility, as a condition co-arising with concentration [12], distinguishes a concentrated mind from an unconcentrated mind [9]. The tranquility that co-arises with concentration has been suggested to make one feel very pleasant [11], thereby further bringing about physiological relaxation [17], which is by far the most researched effect of meditation in Western literature [14]. In fact, one of the intentions of cultivating concentration is to effectively calm the mind and introduce tranquility into the mind [36,37], as concentration is regarded as the primary way to produce a calm mind [17]. In the present study, tranquility is operationally defined as “calmness of the mind”.

Empirically, participants from a wide range of non-clinical [38,39] and clinical populations [40,41] across different age groups [42,43] have been reported to experience calmness in various mindfulness-based programs [44,45]. Tranquility has been proposed to improve the clarity of one’s awareness [17] and to prepare the mind for more expansive practice in open monitoring [6,46]. It also helps buttress the mind against any unsettling experiences that may arise when deeper levels of insight unfold [11] and when participants are having deep explorations into and directly facing their unpleasant experiences in mindfulness-based programs. Tranquility has also been suggested to improve mental health and reduce psychological distress by reducing stress and anxiety [6,14], allowing one to have more control over thoughts [14], helping one to sleep better and to derive new solutions to manage difficult situations [6]. Despite its potential benefits, tranquility as a psychological construct has been rarely studied empirically in mindfulness research.

The cultivation of concentration and tranquility also facilitates the attainment of nonattachment, which is defined as “a flexible, balanced way of relating to one’s experiences without clinging to or suppressing them” [47]. Nonattachment has been demonstrated to be positively associated with [48] and improve mental health [4]. It is proposed that deeper levels of concentration can lead to lower levels of craving and aversion and therefore support the cultivation of nonattachment. In practice, concentration and tranquility can help one to subdue sensations, emotions, and entangling thoughts, and this may also facilitate nonattachment. Reciprocally, when one has stronger nonattachment and can let go more, one can more easily cultivate deeper concentration and tranquility [49].

It is clear that concentration, tranquility, and mindfulness are distinct but closely related constructs, and they may commonly co-arise in mindfulness-based programs and in daily life. Given insufficient empirical emphasis on concentration and tranquility, the present study aimed to examine their relationships with mental health, relative to mindfulness. With no existing self-report measure assessing concentration and tranquility, this study first developed and validated scales measuring the ordinary level of concentration and tranquility, with both scales applicable to the daily life of the general population. The study then examined the cross-sectional and longitudinal relationships of concentration, tranquility, and mindfulness with indicators of mental health. While cross-sectional data could provide preliminary data on how these variables are associated concurrently, longitudinal data allow us to investigate the predictability of concentration, tranquility, and mindfulness on mental health-related variables over time (after controlling for the effects of one another). The following was hypothesized: (a) concentration is positively correlated with attentional control, tranquility, mindfulness, and nonattachment and is negatively correlated with irritability, perceived stress, and psychological distress; (b) tranquility is positively correlated with concentration, attentional control, mindfulness, and nonattachment and is negatively correlated with irritability, perceived stress, and psychological distress; (c) concentration and tranquility can explain perceived stress and psychological distress above and beyond the cross-sectional effect of mindfulness; and (d) longitudinal effect of concentration and tranquility on perceived stress and psychological distress can have a unique contribution above and beyond that of mindfulness.

## 2. Method

### 2.1. Participants

Two samples of participants were collected using convenience sampling in the present study.

Sample 1 was collected in both a university and community in Hong Kong to assess the psychometric properties of the Concentration Scale and Tranquility Scale with exploratory factor analyses (EFA) and confirmatory factor analyses (CFA). The sample consisting of 768 participants was split in half randomly. The first half of 384 participants (58.1% female, mean age = 22.98, *SD* = 6.8, range = 17–62) was used for EFA and the second half of 384 participants (59.4% female, mean age = 23.30, *SD* = 7.5, range = 17–70) was used for CFA. About a third of the participants had completed secondary education (32.8% for EFA; 35.2% for CFA) and two-thirds had completed tertiary education (67.2% for EFA; 64.8% for CFA). Most of them did not have experience in mindfulness practice (78.6% for EFA; 77.9% for CFA).

Sample 2 was collected in university in Hong Kong to examine construct validity and test-retest reliability, the incremental value of concentration and tranquility over the cross-sectional effect of mindfulness on perceived stress and psychological distress, and the incremental value of concentration and tranquility over the longitudinal effect of mindfulness on perceived stress and psychological distress. A total of 333 participants (77.2% female, mean age = 23.40, *SD* = 6.8, range = 18–54) were recruited at baseline, and 268 participants (76.8% female, mean age = 23.50, *SD* = 7.0, range = 18–54) were retained at the two-week follow-up assessment. Around half of the participants had completed secondary education (57.0% for baseline; 56.7% for two-week follow-up) and half had completed tertiary education (42.9% for baseline; 43.3% for two-week follow-up). Most of them did not have experience in mindfulness practice (91.6% for baseline; 91.3% for two-week follow-up).

### 2.2. Procedure

Based on the operational definition of concentration and tranquility, two groups of 31 items in total (16 items for concentration and 15 items for tranquility) were drafted. A focus group of three postgraduate students was asked to review the items to ensure comprehensibility by the general public, and the items were then revised accordingly. The revised items underwent further screening for face validity. Items with content not directly related to the constructs or with highly similar meaning to another item are removed, resulting in a list of 14 items in total (7 items for concentration and 7 items for tranquility). The list of items was sent to a group of experts, including experts in mindfulness, Buddhist scholars, and Buddhist monastics from Hong Kong, Sri Lanka, Taiwan, Thailand, the United Kingdom, and the United States for establishment of content validity. Experts were asked to rate each item based on a 10-point Likert scale from 1 (totally unrelated) to 10 (totally related) according to the relatedness of the content of each item to its corresponding construct. Ratings from 11 experts were obtained and scores of each item were averaged. Items were removed if their average score was lower than 8 out of 10. Data were collected for the abridged sets of 4 items for concentration and 4 items for tranquility.

The study was approved by the Survey and Behavioral Research Ethics Committee at the authors’ institution. Participants from the university were recruited by dissemination of announcements through a centralized email system at the authors’ institution. Participants from the community were recruited by dissemination of announcements through social media. The inclusion criteria for participants were as follows: (i) having access to the Internet and (ii) being 17 years old or older. There were no exclusion criteria for this study. An email containing the link to an online questionnaire was sent to each participant after online registration. The participants completed the online questionnaire after reading a description of the study and giving their informed consent. Participants’ completion time of the questionnaire in Sample 1 was around 7 min. The completion time for the first questionnaire for Sample 2 was around 30 min, given it contained other measures for construct validity and hierarchical multiple regression. The approximate time taken to complete the second questionnaire for Sample 2 for test-retest reliability and longitudinal investigation of incremental value was about 15 min, given it contained fewer measures as compared with that for construct validity in the first questionnaire. For participants in Sample 2, a subsequent email with unique link (to enable trackable identities) to the second online questionnaire was sent to them for completion two weeks after completion of the first questionnaire. A total of 268 participants successfully completed the second questionnaire. HKD 70 was given to them after completion of all required online questionnaires as reward for their participation.

### 2.3. Measures

**Concentration** The 4-item Concentration Scale was used to measure concentration on a 6-point Likert-type scale from 1 (never) to 6 (always). Higher score indicated higher level of concentration. Good internal consistency was found (Cronbach’s alpha = 0.80; omega = 0.80) in the present study. The questionnaire to be used for the Concentration Scale is listed in the Supplementary Materials.

**Tranquility** The 4-item Tranquility Scale was used to measure tranquility on a 6-point Likert-type scale from 1 (very untrue of me) to 6 (very true of me) for 3 items and from 1 (never) to 6 (always) for 1 item. Higher score indicated higher level of tranquility. It showed satisfactory internal consistency (Cronbach’s alpha = 0.75; omega = 0.75) in the present study. The questionnaire to be used for the Tranquility Scale is listed in the Supplementary Materials.

**Attentional Control** The 20-item Attentional Control Scale [50] measures one’s ability to control attention on a 4-point Likert-type scale from 1 (almost never) to 4 (always). A higher score indicated higher ability in attentional control. In previous research, it has shown satisfactory internal consistency (Cronbach’s alpha = 0.84) and validity [51]. Satisfactory internal consistency (Cronbach’s alpha = 0.78; omega = 0.76) was observed in the present study.

**Irritability** The 5-item Brief Irritability Scale [52] was used to measure irritability, the proneness and susceptibility to states of frustration and annoyance despite little provocation on a 6-point Likert-type scale from 1 (never) to 6 (always). A higher score indicated higher level of irritability. It has shown good internal consistency (Cronbach’s alpha = 0.88) and validity in previous research [52], and it had good internal consistency (Cronbach’s alpha = 0.89; omega = 0.90) in the present study.

**Mindfulness** The “acting with awareness”, “observing”, and “describing” subscales of the Five-Facet Mindfulness Questionnaire—Short Form [53] were used to measure mindfulness on a 5-point Likert-type scale from 1 (never or very rarely true) to 5 (very often or always true), with higher score of the composite score indicating higher level of mindfulness. The “nonjudging” subscale was excluded because nonjudging was arguably either contradictory to another important aspect of practicing mindfulness—discernment and clear comprehension [13,29], or representing the absence of judgmental reactivity [54]—and thus may belong to another distinct psychological construct—equanimity [55]. The “non-reacting” subscale was excluded because it conceptually belonged to equanimity [56]. Such categorization of the subscales of “acting with awareness”, “observing”, and “describing” as mindfulness was also adopted by Hadash et al. [57]. The three subscales have shown good validity and internal consistency in previous research [53], and good internal consistency of their composite score was found (Cronbach’s alpha = 0.80; omega = 0.89) in the present study.

**Nonattachment** The 8-item Nonattachment Scale—Short Form [58], was used to measure the level of nonattachment—being flexible and balanced when relating to one’s experiences without clinging to or suppressing them—using a 6-point Likert-type scale from 1 (disagree strongly) to 6 (agree strongly). Higher score indicates higher level of nonattachment. It has shown good validity in previous research [58] and had excellent internal consistency (Cronbach’s alpha = 0.90; omega = 0.90) in the present study.

**Perceived Stress** The 10-item Perceived Stress Scale [59] measures perceived stress using a 5-point Likert-type scale from 1 (never) to 5 (very often). A higher score indicates higher level of perceived stress. It has shown good internal consistency (Cronbach’s alpha = 0.83) and good concurrent validity in previous research [60] and had good internal consistency (Cronbach’s alpha = 0.84; omega = 0.85) in the present study.

**Psychological Distress** The 10-item Kessler Psychological Distress Scale [61] measures nonspecific psychological distress experienced using a 5-point Likert-type scale from 1 (none of the time) to 5 (all of the time). A higher score indicates higher level of psychological distress. It has been validated among people with common mental disorders [61] and had excellent internal consistency (Cronbach’s alpha = 0.91; omega = 0.92) in the present study.

### 2.4. Data Analyses

Statistical Software R of version 3.5.0 with package “Psych” of version 1.8.3.3 [62] and “Lavaan” of version 0.6-3 [63] was used.

#### 2.4.1. Exploratory Factor Analyses

To examine the factor structure of the Concentration Scale and Tranquility Scale and remove any item with factor loading lower than 0.4 [64,65], exploratory factor analyses using principal axis factoring analysis with oblimin rotation were conducted on the first half of Sample 1 (*n* = 384). The number of factors to be retained was determined by parallel analysis [66].

#### 2.4.2. Confirmatory Factor Analyses

**Figure 1:**
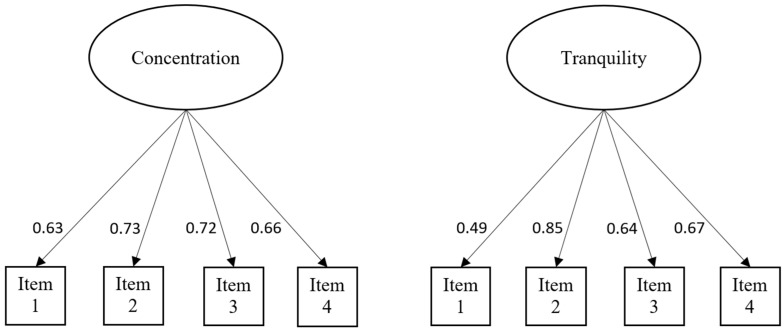
Results of confirmatory factor analysis of the Concentration Scale and Tranquility Scale.

Confirmatory factor analyses adopting single factor model (see Figure 1) were then performed on the second half of Sample 1 (*n* = 384). Given that evaluation with at least two model fit indices is considered to be good criteria for model evaluation [67], results were evaluated using the following model fit indices: comparative fit index (CFI), Tucker–Lewis index (TLI), and standardized root mean square residual (SRMR). Root mean square error of approximation (RMSEA) is recommended not to be used for models with small degree of freedom due to its suboptimal performance and potential false indication as poor fitting model for models with small degree of freedom [68]. Therefore, RMSEA was not used because the single-factor models of Concentration Scale and Tranquility Scale have degree of freedom of 2 only. CFI value greater than 0.9, TLI value greater than 0.9, and SRMR value less than 0.09 indicate an acceptable fit of the model [67].

#### 2.4.3. Study of Test-Retest Reliability and Construct Validity

Responses from participants in Sample 2 were analyzed to study test-retest reliability and construct validity of the Concentration Scale and Tranquility Scale. Test-retest reliability of both scales was established by assessing Pearson’s correlations between the scores across two weeks. Pearson’s correlations of the Concentration Scale and the Tranquility Scale with variables that are related to (1) concentration-related concept (i.e., attention control); (2) tranquility-related concept (i.e., irritability); (3) mindfulness and related concept (i.e., mindfulness and nonattachment); and (4) perceived stress and psychological distress were examined to establish the construct validity of both scales.

#### 2.4.4. Hierarchical Multiple Regression Analysis on Cross-Sectional Data

Hierarchical multiple regression analysis [69] on cross-sectional data was used to investigate incremental value of concentration and tranquility over the cross-sectional effect of mindfulness on the outcome variables (i.e., perceived stress and psychological distress). In the first analysis, demographic characteristics were put into the first block, and mindfulness was put into the second block. In the second analysis, demographic characteristics and mindfulness were put into the first block, and concentration was put into the second block. In the third analysis, demographic characteristics and mindfulness were put into the first block, and tranquility was put into the second block. In the fourth analysis, demographic characteristics and mindfulness were put into the first block, and concentration and tranquility were put into the second block. Incremental values of concentration and tranquility over cross-sectional effect of mindfulness on the outcome variables were reflected by change in values of R^2^.

#### 2.4.5. Hierarchical Multiple Regression Analysis on Longitudinal Data

Hierarchical multiple regression analysis on longitudinal data with two time points across two weeks was used to investigate the incremental value of concentration and tranquility over the longitudinal effect of mindfulness on the outcome variables (i.e., perceived stress and psychological distress). In the first analysis, demographic characteristics and outcome variables at baseline (T1) were put into the first block, and T1 mindfulness was put into the second block. In the second analysis, demographic characteristics, T1 mindfulness, and T1 outcome variables were put into the first block, and T1 concentration was put into the second block. In the third analysis, demographic characteristics, T1 outcome variables, and T1 mindfulness were put into the first block, and T1 tranquility was put into the second block. In the fourth analysis, demographic characteristics, T1 outcome variables, and T1 mindfulness were put into the first block, and T1 concentration and T1 tranquility were put into the second block. Across all the analyses, T2 outcome variables were entered as dependent variables. Incremental values of T1 concentration and T1 tranquility on T2 outcome variables, after controlling for the effect of T1 mindfulness, T1 outcome variables, and demographic characteristics, were reflected by change in values of R^2^.

## 3. Results

### 3.1. Scale Validation

#### 3.1.1. Exploratory Factor Analyses

In the EFA (see Table 1), one-factor solutions were suggested for both scales by the results of the parallel analyses. No items were removed as the factor loadings for all the items were higher than 0.4. The single factor of the Concentration Scale explained 49.8% of the variance and its internal consistency was reflected in its Cronbach’s alpha, 0.80, and omega, 0.80. The single factor of the Tranquility Scale explained 44.6% of the variance, and its Cronbach’s alpha was 0.75 and omega was 0.75.

#### 3.1.2. Confirmatory Factor Analyses

In the CFA (see Figure 1), the results showed that the models for the Concentration Scale and Tranquility Scale had an excellent fit to the data (concentration: CFI = 0.98, TLI = 0.94, SRMR = 0.03; tranquility: CFI = 1.00, TLI = 1.00, SRMR = 0.01).

#### 3.1.3. Study of Test-Retest Reliability and Construct Validity

Both scales demonstrated satisfactory test-retest reliability (concentration: *r* = 0.71, *p* < 0.001; and tranquility: *r* = 0.75, *p* < 0.001) over two weeks.

In general, the results (see Table 2) supported our hypotheses, such that the concentration composite score had a strong correlation with concentration-related concept (|*r*| = 0.58), moderate to strong correlations with tranquility and tranquility-related concept (|*r*s| = 0.36 to 0.55), moderate to strong correlations with mindfulness and related concept (|*r*s| = 0.36 to 0.50), and moderate correlations with perceived stress and psychological distress (|*r*s| = 0.34 to 0.41). The tranquility composite score had moderate to strong correlations with concentration and concentration-related concept (|*r*s| = 0.40 to 0.55), a moderate correlation with tranquility-related concept (|*r*| = 0.41), moderate to strong correlations with mindfulness and related concept (|*r*s| = 0.40 to 0.64), and moderate to strong correlations with perceived stress and psychological distress (|*r*s| = 0.47 to 0.53). The correlations were all in line with the expected directions, as hypothesized.

### 3.2. Study of Incremental Value over Mindfulness on Mental Health

#### 3.2.1. Hierarchical Multiple Regression Analyses on Cross-Sectional Data

In the hierarchical multiple regression analyses on cross-sectional data (see Table 3), the results of the first analysis showed that the associations between mindfulness and all the outcome variables were significant (βs = −0.34 to −0.23, *p*s < 0.001). In the second analysis, the associations between concentration and all the outcome variables, after controlling for mindfulness, were significant (βs = −0.33 to −0.30, *p*s < 0.001), though associations of mindfulness with psychological distress were not significant. The incremental value (as reflected by the change in the value of *R*^2^) of concentration over mindfulness on the outcome variables ranged from 6 to 8%. The associations of concentration with all the outcome variables were higher in value than those of mindfulness. In the third analysis, the associations between tranquility and all the outcome variables, after controlling for mindfulness, were significant (βs = −0.48 to –0.47, *p*s < 0.001), though associations of mindfulness with psychological distress were not significant. The incremental value of tranquility over mindfulness on the outcome variables ranged from 18 to 19%. The associations of tranquility with all outcome variables were higher in value than those of mindfulness. When both concentration and tranquility were added to the second block in the fourth analysis, the associations of mindfulness with all outcome variables were not significant, and the associations of concentration with psychological distress were not significant, while the associations between tranquility and all the outcome variables were significant (βs = −0.43, *p*s < 0.001). The incremental value of both concentration and tranquility over mindfulness on all the variables ranged from 19 to 20%.

#### 3.2.2. Hierarchical Multiple Regression Analyses on Longitudinal Data

In the hierarchical multiple regression analyses on longitudinal data (see Table 4), the results of the first analysis indicated that the associations between T1 mindfulness and all T2 outcome variables were not significant after controlling for the T1 outcome variables and demographic characteristics. In the second analysis, after controlling for T1 mindfulness, T1 outcome variables, and demographic characteristics, the associations between T1 concentration and all the T2 outcome variables were not significant. In the third analysis, after controlling for T1 mindfulness, T1 outcome variables, and demographic characteristics, the associations between T1 tranquility and all the T2 outcome variables were significant (βs = −0.22 to −0.13, *p*s < 0.05), and the incremental values of T1 tranquility on the T2 outcome variables were significant (Δ*R*^2^s = 0.01 to 0.03, *p*s < 0.05). When both T1 concentration and T1 tranquility were added to the second block in the fourth analysis, the associations between T1 concentration and all T2 outcome variables were not significant, while the associations between T1 tranquility and all T2 outcome variables were significant (βs = −0.23 to −0.12, *p*s < 0.05).

## 4. Discussion

The present study examined how concentration and tranquility might have an incremental value over mindfulness on outcome variables concerning mental health. With no existing self-report measure assessing concentration and tranquility, the present study first developed and validated the Concentration Scale and Tranquility Scale, both of which are applicable to the daily life of the general population. Both scales were shown to have satisfactory construct validity, internal consistency, and test-retest reliability. Our results also supported the incremental value of concentration and tranquility on perceived stress and psychological distress above and beyond the effect of mindfulness cross-sectionally. Our findings also supported the longitudinal effects of tranquility on perceived stress and psychological distress.

Consistent with our hypotheses, the associations of concentration and tranquility with other measures were significant in the hypothesized directions with moderate to strong correlations. The correlations between concentration, tranquility, and mindfulness were moderate to strong, and this is consistent with their relationship in that they are very intertwined [14,70] and mutually reinforce each other [14,30]. Tranquility, as a condition co-arising with concentration [12], was also shown to have a strong correlation with concentration. Future interventional studies can empirically investigate the relationships of co-arising and mutual causation among concentration, tranquility, and mindfulness.

The cross-sectional results of the hierarchical regression suggested that concentration and tranquility might have unique contributions to perceived stress and psychological distress above and beyond mindfulness, as hypothesized. Our results found that after the effect of concentration was accounted for, the association of mindfulness with psychological distress was no longer significant. On the contrary, concentration, after accounting for the effect of mindfulness, was significantly associated with all of the outcome variables. Although both mindfulness and concentration may reduce mental proliferation [36] and thus improve mental health, the results showed that concentration has a stronger effect than mindfulness on perceived stress and psychological distress in the current sample which was mainly composed of non-meditators. This provided preliminary evidence of the incremental role of concentration on mental health, in addition to mindfulness. Future interventional research should investigate the relative effects of concentration and mindfulness on mental health and the mechanisms through which they produce these effects. After tranquility was further added in the fourth analysis, the association of concentration with psychological distress was no longer significant, whereas the associations of tranquility with all the outcome variables were significant, after accounting for the effects of both concentration and mindfulness. This observation may be explained by the possibility that, for the majority of participants in the current sample who were non-meditators and had never meditated, tranquility was likely to be more soothing and comprehensible than being aware (mindful) and sustainedly attentive (concentrative), and thus can better explain mental health.

Differing from the cross-sectional results, the longitudinal findings showed that mindfulness or concentration at baseline could not predict any outcome variable concerning perceived stress and psychological distress at the two-week follow-up assessment, after accounting for the effect of outcome variables at baseline and demographic characteristics. Tranquility, however, could significantly predict perceived stress and psychological distress at the two-week follow-up assessment. Consistent with previous speculation, for the majority in the current sample who had never meditated, tranquility was likely to be more soothing and comprehensible than being aware and sustainedly attentive, and therefore it could better predict mental health in the two-week time frame. Future research can examine the relationships of these variables for both non-meditator and meditator samples to explore possible differences and commonalities.

All in all, the cross-sectional and longitudinal evidence supported the hypotheses that concentration and tranquility have a unique contribution towards explaining perceived stress and psychological distress above and beyond that of mindfulness. It also highlighted the importance of separately accounting for the effect of concentration and tranquility—the two constructs that are commonly conflated with but distinct from mindfulness—in mindfulness research.

Concentration and tranquility often co-arise with mindfulness during meditation and mindfulness practice, and they are commonly mixed up with mindfulness by participants of mindfulness-based programs even though they are distinct concepts. However, they have rarely been empirically investigated in previous studies due to the absence of validated measures. The present study developed and validated two short measures that provide options for future researchers and practitioners to empirically and systematically measure concentration and tranquility alongside with other mindfulness-related psychological concepts. This way, researchers can directly compare and more comprehensively understand how these concepts contribute to individuals’ mental health in mindfulness practice and daily life. This direct investigation and comparison also provide important data for practitioners and scholars to consider explicitly introducing or teaching concentration and tranquility and related practices to individuals to promote better understanding of these commonly confused psychological constructs and facilitate their cultivation and development in daily life.

### Limitations and Future Research

The present study has several limitations and we would like to provide suggestions for future research studies. Samples from the university and the community, with the majority having no experience in mindfulness or meditation practice, were used in the present study. Given that levels of concentration, mindfulness, and tranquility can be influenced by one’s experience in practicing meditation, future studies should also validate the Concentration Scale and Tranquility Scale and replicate our findings in meditator samples. Moreover, the present study is non-experimental in nature. Although a longitudinal study design, complemented with a cross-sectional study design, was employed to investigate the temporal relationships between the variables over two weeks, causality could not be established. Future interventional studies can investigate whether concentration and tranquility can be cultivated alongside with mindfulness by mindfulness-based programs and how they may affect mental health, after accounting for one another, and ultimately pave the way for the curricula of mindfulness-based programs to cover the concepts of concentration and tranquility. In addition, the concentration construct in the current study focuses on the ordinary level of concentration, and it does not cover all features of concentration arising from deeper levels of absorption which rarely arise in daily life. Future studies may explore the feasibility of developing a measure covering deeper levels of absorption in concentration. Furthermore, the current study developed the trait measures of concentration and tranquility which can be used in empirical research on the general population to capture these constructs in people’s daily lives. Future studies may develop state measures on concentration and tranquility as well to assess momentary changes in concentration and tranquility upon meditative practice.

## 5. Conclusions

The present study demonstrated the incremental value of concentration and tranquility—constructs that have been commonly neglected and conflated with mindfulness both in empirical research and in mindfulness practice—over mindfulness on mental health. Having developed empirical measures of concentration and tranquility in the present study, these constructs can be explicitly investigated along with mindfulness in future mindfulness-based programs, the daily lives of the general population, and specific settings (e.g., schools and health care units) to study their potential effects on mental health and to provide us with a broader picture on how mindfulness practices benefit human wellness.

## Figures and Tables

**Table 1:** Factor loadings of the final Concentration Scale and final Tranquility Scale.

Items	Factor Loadings
Concentration Scale	
1. When listening to a person speaking, I am not distracted by things going on around me (e.g., noise).	0.73
2. When doing a task, I stay focused on what I am doing.	0.64
3. When doing a task, I am not distracted by things going on around me (e.g., noise).	0.78
4. When listening to a person speaking, I stay focused on listening.	0.66
Tranquility Scale	
1. When I face unpleasant encounter, I cannot calm myself down. (Reversed Question)	0.50
2. I can stabilize my emotion to create a feeling of calmness.	0.77
3. When I face unpredictable future outcomes, I can calm myself down.	0.71
4. I feel calm and at ease.	0.67

**Table 2:** Means, standard deviations, and bivariate correlations among all variables.

	1	2	3	4	5	6	7	8
Concentration and related concept								
1. Concentration	-	0.58	0.55	−0.36	0.50	0.36	−0.41	−0.34
2. Attentional control		-	0.40	−0.29	0.47	0.28	−0.32	−0.27
Tranquility and related concept								
3. Tranquility			-	−0.41	0.40	0.64	−0.53	−0.47
4. Irritability				-	−0.22	−0.34	0.57	0.50
Mindfulness and related concept								
5. Mindfulness					-	0.26	−0.35	−0.25
6. Nonattachment						-	−0.53	−0.46
Mental health indicators								
7. Perceived stress							-	0.73
8. Psychological distress								-
Mean	3.98	2.56	3.70	2.57	3.08	3.71	3.11	2.52
Standard Deviation	0.83	0.32	0.71	0.88	0.52	0.82	0.52	0.71

Note. All correlations are significant at the 0.001 level.

**Table 3:** Incremental value of concentration and tranquility over the cross-sectional effect of mindfulness on outcome variables: results of cross-sectional hierarchical multiple regression analyses (*n* = 333).

Outcome Variables	First Analysis			Second Analysis				Third Analysis				Fourth Analysis				
	MF β	*R* ^2^	Δ*R*^2^	MF β	CON β	*R* ^2^	Δ*R*^2^	MF β	TRAN β	*R* ^2^	Δ*R*^2^	MF β	CON β	TRAN β	*R* ^2^	Δ*R*^2^
Perceived stress	−0.34 ***	0.12	0.11 ***	−0.17 **	−0.33 ***	0.19	0.08 ***	−0.14 **	−0.48 ***	0.31	0.19 ***	−0.09	−0.13 *	−0.43 ***	0.32	0.20 ***
Psychological distress	−0.23 ***	0.07	0.05 ***	−0.08	−0.30 ***	0.13	0.06 ***	−0.03	−0.47 ***	0.26	0.18 ***	−0.00	−0.09	−0.43 ***	0.26	0.19 ***

Gender and age were controlled but not shown in the table, *MF* = mindfulness; *CON* = concentration; *TRAN* = tranquility, * *p* < 0.05; ** *p* < 0.01; *** *p* < 0.001.

**Table 4:** Longitudinal effect of mindfulness, concentration and tranquility on outcome variables: results of longitudinal hierarchical multiple regression analyses (n = 268).

T2 Outcome Variables	First Analysis				Second Analysis				Third Analysis				Fourth Analysis				
	T1 OV β	MF β	*R* ^2^	Δ*R*^2^	MF β	CON β	*R* ^2^	Δ*R*^2^	MF β	TRAN β	*R* ^2^	Δ*R*^2^	MF β	CON β	TRAN β	*R* ^2^	Δ*R*^2^
Perceived stress	0.73 ***	−0.01	0.57	0.00	0.03	−0.08	0.57	0.00	0.05	−0.22 ***	0.60	0.03 ***	0.05	0.01	−0.23 ***	0.60	0.03 ***
Psychological distress	0.77 ***	−0.03	0.60	0.00	−0.01	−0.06	0.60	0.00	0.01	−0.13 *	0.61	0.01 *	0.01	−0.02	−0.12 *	0.61	0.01 *

Gender and age were controlled but not shown in the table, *T1 OV* = outcome variable at T1; *MF* = mindfulness; *CON* = concentration; *TRAN* = tranquility, * *p* < 0.05; *** *p* < 0.001.

## Data Availability

The data presented in this study are available on request from the corresponding author.

## Supplementary Materials

The following supporting information can be downloaded at: https://www.mdpi.com/article/10.3390/ijerph20085470/s1, Table S1: Questionnaire to be Used for the Concentration Scale and Tranquility Scale.
