# Supplementary material for: Going beyond Mindfulness: How Concentration and Tranquility Commonly Co-Arising with Mindfulness Account for Mental Health
Source: Int J Environ Res Public Health. 2023 Apr 11;20(8):5470. doi: 10.3390/ijerph20085470 (PMC10138238; doi:10.3390/ijerph20085470)
Supplement: Supplementary file 1 [file ijerph-20-05470-s001.zip › ijerph-2240731-supplementary.pdf]

Table S1. Questionnaire to be Used for the Concentration Scale and Tranquility Scale.

Concentration Scale						
How often were you in the following mental state (if the following situation happened)?	1 = never	2 = seldom	3 = occasionally	4 = sometimes	5 = often	6 = always
1. When listening to a person speaking, I am not distracted by things going on around me (e.g., noise).<br>2. When doing a task, I stay focused on what I am doing.<br>3. When doing a task, I am not distracted by things going on around me (e.g., noise).<br>4. When listening to a person speaking, I stay focused on listening.						
**Tranquility Scale**						
To what extent do you agree with the following description about yourself?	1 = very untrue of me	2 = untrue of me	3 = somewhat untrue of me	4 = somewhat true of me	5 = true of me	6 = very true of me
1. When I face unpleasant encounter, I cannot calm myself down. *<br>2. I can stabilize my emotion to create a feeling of calmness.<br>3. When I face unpredictable future outcomes, I can calm myself down.						
How often were you in the following mental state?	1 = never	2 = seldom	3 = occasionally	4 = sometimes	5 = often	6 = always
4. I feel calm and at ease.						

* Reversed question.
